# Inequalities in financial risk protection in Bangladesh: an assessment of universal health coverage

**DOI:** 10.1186/s12939-017-0556-4

**Published:** 2017-04-04

**Authors:** Md. Rashedul Islam, Md. Shafiur Rahman, Zobida Islam, Cherri Zhang B. Nurs, Papia Sultana, Md. Mizanur Rahman

**Affiliations:** 1 Department of Computer Science, Uttara Commerce College, Dhaka, Bangladesh; 2 Department of Global Health Policy, The University of Tokyo, Tokyo, Japan; 3 Department of Public Health, First Capital University of Bangladesh, Chuadanga, Bangladesh; 4 Department of Statistics, University of Rajshahi, Rajshahi, Bangladesh; 5 Department of Population Science and Human Resource Development, University of Rajshahi, Rajshahi, 6205 Bangladesh

**Keywords:** OOP payment, Catastrophic expenditure, Impoverishment, Hardship financing, Universal health coverage, Inequalities, Bangladesh

## Abstract

**Background:**

Financial risk protection and equity are major components of universal health coverage (UHC), which is defined as ensuring access to health services for all citizens without any undue financial burden. We investigated progress towards UHC financial risk indicators and assessed variability of inequalities in financial risk protection indicators by wealth quintile. We further examined the determinants of different financial hardship indicators related to healthcare costs.

**Methods:**

A cross-sectional, three-stage probability survey was conducted in Bangladesh, which collected information from 1600 households from August to November 2011. Catastrophic health payments, impoverishment, and distress financing (borrowing or selling assets) were treated as financial hardship indicators in UHC. Poisson regression models were used to identify the determinants of catastrophic payment, impoverishment and distress financing separately. Slope, relative and concentration indices of inequalities were used to assess wealth-based inequalities in financial hardship indicators.

**Results:**

The study found that around 9% of households incurred catastrophic payments, 7% faced distress financing, and 6% experienced impoverishing health payments in Bangladesh. Slope index of inequality indicated that the incidence of catastrophic health payment and distress financing among the richest households were 12 and 9 percentage points lower than the poorest households respectively. Multivariable Poisson regression models revealed that all UHC financial hardship indicators were significantly higher among household that had members who received inpatient care or were in the poorest quintile. The presence of a member with chronic illness in a household increased the risk of impoverishment by nearly double.

**Conclusion:**

This study identified a greater inequality in UHC financial hardship indicators. Rich households in Bangladesh were facing disproportionately less financial hardship than the poor ones. Households can be protected from financial hardship associated with healthcare costs by implementing risk pooling mechanism, increasing GDP spending on health, and properly monitoring subsidized programs in public health facilities.

**Electronic supplementary material:**

The online version of this article (doi:10.1186/s12939-017-0556-4) contains supplementary material, which is available to authorized users.

## Background

Achieving universal health coverage (UHC) is one of the key targets in the proposed Sustainable Development Goals (SDGs) [1]. There are two key targets in UHC plan: having at least 80% essential health service coverage, and 100% financial risk protection from catastrophic and impoverishing payment for health services by 2030 [2, 3]. The World Health Organization (WHO) and World Bank (WB) jointly developed a framework for assessing UHC through three dimensions: population, health service coverage, and proportion of health expenditure covered by formal risk pooling mechanisms [2]. Financial risk protection plan is now accepted as a key mechanism to ensure affordable and equitable access to care for all citizens of a country irrespective of their socio-economic statuses [4]. Many countries adopted UHC as a top priority for their national health systems in order to alleviate poverty and improve health outcomes through ensuring equity in access to care [2].

Similar to other South Asian countries, Bangladesh is simultaneously experiencing a double burden of diseases, low health service coverage, and a lack of financial risk protection mechanism in their health system [5, 6]. Bangladesh has a dual healthcare systems, with both public and private health services co-existing in most areas. There are three main levels: primary health care, district, and divisional or tertiary levels [7]. The public sector is largely used for outpatient, inpatient, and preventive care, while the private sector is used mainly for outpatient and inpatient curative care. The main public health provider is the Ministry of Health and Family Welfare (MOHFW), which provides primary, secondary and tertiary care through various types of health facilities (such as general hospitals, district hospitals and health clinics). Public health services are heavily subsidized by the government, and primary care services at health clinics are delivered at almost free of charge, with each patient being charged a nominal fee of Bangladesh Taka13 (equivalent to US$ 0.17 in 2011) for each outpatient visit [8]. Secondary and tertiary care services provided at hospital facilities are also highly subsidized by the government. Private health providers, complementing the medical services provided by the government, mainly focuses on curative services including general practitioner clinics, medical centers, and private hospitals. Bangladesh currently has neither a national health insurance scheme nor a well-developed private insurance market [9]. There are a number of small scale NGO-based community insurance schemes, often operating in conjunction with micro-financing schemes, but these cover less than 1% of the total population and target mainly poor populations [9].

Health financing is underfunded in Bangladesh; government spend less than one percent of gross domestic product (GDP) on health which is the lowest among South Asian countries [10].

Health sector is also neglected in terms of country’s total budget, only 4.3% of the total budget were allocated for the health sector in financial year 2015–16 [11]. Out-of-pocket (OOP) payment remain the main source of healthcare funding in Bangladesh, making up 63.3% of total healthcare expenditure [9]. Inequality is another concern in countries with fragile health systems like Bangladesh, and disadvantaged populations are often restricted in their financial access to healthcare services. Inadequate public funding for health services, limited access to health insurance plans, and unexpected OOP payments can trigger asset depletion, indebtedness, and reductions in essential consumption, which in turn prevent access to health services and may ultimately lead to financial catastrophe, distress financing, and impoverishment [12–19].

In order to measure and track Bangladesh’s progress towards UHC and its financial risk protection indicators, we assessed incidence of catastrophic and impoverishing health expenditure and distress financing associated with OOP payments. We further examined the determinants of different financial hardship indicators related to healthcare costs using representative survey data.

## Methods

### Study area and design

This study took place in Rajshahi city of Bangladesh, the third largest city located in the north-western part of the country. Rajshahi district has a population of 2.6 million, with an average household size of approximately four people [20], and broadly represent many urban areas in Bangladesh based on demographic distribution [20]. The literacy rate is 71 and 62% for males and females, respectively. This was a cross-sectional study based on a three-stage, cluster-sampling methodology, which collected information from 1600 households from August to November 2011. The overall response rate was 99.6%.

### Data collection

Interviewers recorded information on household member’s socio-demographic characteristics, and household consumption or expenditure in the past 30 days or past 12 months using a structured questionnaire from household heads after obtaining informed consent. The study used a recall period for all illnesses in the past 30 days and at least 3 months’ duration for chronic diseases in the year prior to interview. A condition was considered chronic if it lasted or was expected to last for more than 3 months [21]. Data were collected on the onset or duration of illness, diagnosis, treatment response, and cost and coping strategies separately. Respondents were asked about their main symptoms and (eventual) diagnoses followed by whether the diagnosis was made by professional medical doctors, i.e. MBBS doctors.

### Measurement of outcomes

In line with other studies, financial risk protection coverage was assessed from incidence of catastrophic and impoverishing health payments [22]. Additionally, incidence of distress financing resulting from OOP was estimated to understand the coping strategy. A household’s expenditure was treated as catastrophic if it exceeded 40% of household capacity to pay [23, 24]. Household capacity to pay refers to the effective income remaining after meeting basic needs that is non-subsistence spending. Subsistence expenditure for each household was estimated by multiplying poverty line with the equivalent household size. A household’s health expenditure was treated as impoverishing when its total per capita consumption spending fell below the poverty line after paying for health care. We estimated the poverty line based on subsistence food expenditure as proposed by World Health Organization [24, 25]. The poverty line was determined based on the average food consumption at the 45th and 55th percentiles of the total household expenditure of the sampled household. Household consumption expenditure was estimated following the standard guidelines [26]. Distress financing involves funding for healthcare costs by borrowing money from relatives or bank and selling household assets [16, 27].

### Covariates

In this study, the average number of children and adults per household, presence of household member aged over 65 years, presence of chronic illness in any member of the household, care-seeking behavior, household consumption quintile, household size, and household head educational status were considered as covariates.

### Statistical analysis

Descriptive statistics were calculated using the mean (confidence interval) or frequency and proportions as appropriate. Poisson regression was used to identify the determinants of catastrophic health expenditure, impoverishment, and distress financing. For equity analysis, socio-economic status of each household was assessed based on household total consumption expenditure. Households were ranked in ascending order based on per capita total consumption expenditure, and divided into quintiles, with quintile 1 (Q1) as the poorest 20% of households and quintile 5 (Q5) representing the richest. We assessed both absolute and relative measures of equity. The slope index of inequality [SII] was used as an absolute measure of inequality, whereas the relative index of inequality [RII] and the concentration index were used as relative measures of inequality [28]. The main purpose of absolute index of inequalities is to interpret the difference in coverage between the extreme wealth quintiles (Q5-Q1). The SII reflects the difference in coverage values in percentage points between individuals at the top and bottom of the wealth scales. We calculated the SII and RII by regressing financial hardship indicators against the household’s relative rank in the cumulative distribution of wealth position. The concentration index indicates the magnitude of relative inequality [28–30]. This index produced values that ranged from −1 to 1. When the concentration index value is zero there is no inequality i.e. no difference in financial burden between poor and rich populations. A negative value indicates the poor population is incurring more financial burden, while a positive value indicates the rich population is facing more financial burden. All analyses were adjusted for the probability sample design. Data management and analysis was performed in Stata/MP Version 14.0.

## Results

### Background characteristics

The average household size in the sample of Rajshahi city was 4.6 (95% CI: 4.5–4.7) and the average number of dependent members was 2.0 (95% CI: 2.0–2.1) per household (Additional file 1: Table S1). The average number of illnesses was 2.8 per household (95% CI: 2.6–2.9). About 71.5% (95% CI: 67.4–75.2) of households had at least one chronic illness in the past 12 months prior to interview. Of the 1593 completed households, 92% incurred health expenditure in the past 30 days recall period. The socio-demographic characteristics of our study population are presented in Table 1.

**Table 1:** Descriptive statistics of households and household heads, Bangladesh, 2011

Household characteristics	Frequency	Percentage	95% CI
Gender of household head			
Male	1447	90.5	88.6–92.1
Female	146	9.5	7.9–11.4
Educational status of household head			
No education	258	17.1	13.9–20.8
Primary	310	20.8	17.4–24.7
Secondary	420	27.2	24.5–30.2
Higher	605	34.9	29.2–41.1
Household member over 65 years			
Yes	136	8.5	7.0–10.2
No	1457	91.6	89.8–93.1
Presence of illness in the last 30 days			
Yes	1501	93.7	91.4–95.3
No	92	6.3	4.8–8.3
Member with chronic disease			
Yes	1148	71.5	67.4–75.2
No	445	28.5	24.8–32.6
Utilization of health services			
Inpatient	65	4.3	3.21–5.6
Outpatient public	253	16.1	13.6–18.9
Outpatient private	385	22.8	19.7–26.2
Outpatient public and private	105	6.3	4.7–8.4
Self-medication/traditional healer	785	50.6	45.8–55.3

*CI* confidence interval

### Equity in financial hardship indicators

**Fig. 1:**
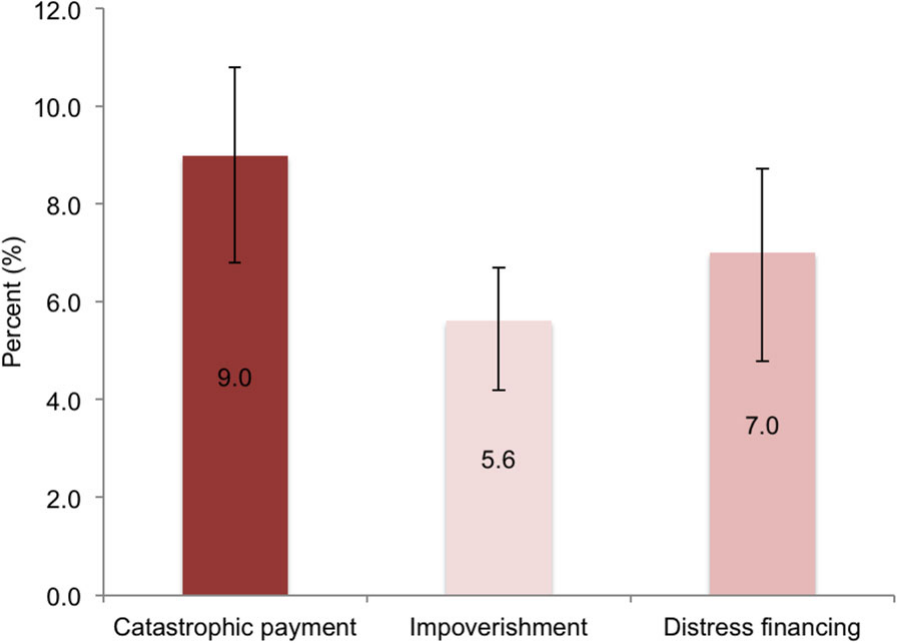
Proportion of household incur financial hardship related to healthcare costs, Bangladesh, 2011

**Fig. 2:**
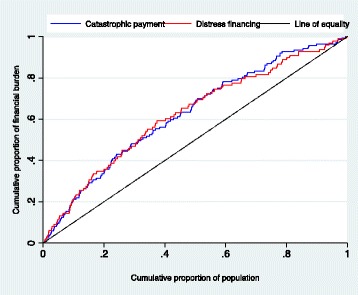
Concentration curve for catastrophic payment and distress financing related to health care costs, Bangladesh, 2011

Around 9.0% (95% CI: 7.2–11.2) of households incurred catastrophic health payment, 5.6% (95% CI: 4.5–7.0) of households experienced impoverishing health expenditure, and 7.0% (95% CI: 5.3–9.2) faced distress financing to pay for health care costs (Fig. 1). Detailed proportion of financial hardship by different socio-demographic characteristics is presented in the Additional file 1 (Table S2). Concentration curves for catastrophic payment and distress financing both lie above the line of equality, indicating a disproportionately higher concentration of catastrophic payment and distress financing in poor households than in rich ones (Fig. 2). Significant differences in catastrophic payment and distress financing among poor and rich households were found in all three measures of inequality indices (Table 2). The SII indicated that incidence of catastrophic health payment and distress financing among the richest households were 12 and 9 percentage points lower than poorest households respectively (Table 2).

**Table 2:** Inequalities in catastrophic payment and distress financing related to health care costs, Bangladesh, 2011

	Catastrophic payments	Distress financing
Household consumption quintile, % (95% CI)		
Quintile 1 (poorest)	14.3 (10.3–19.6)	11.7 (8.3–16.4)
Quintile 2	9.7 (6.2–15.0)	7.6 (4.9–11.7)
Quintile 3	9.2 (5.7–14.5)	6.1 (3.1–11.8)
Quintile 4	7.1 (4.3–11.4)	4.8 (2.6–8.6)
Quintile 5 (richest)	3.4 (1.7–6.4)	3.9 (2.0–7.7)
Inequality index, (95% CI)		
Slope index of inequality (Q5-Q1)	−12.0 (−18.6 to −5.4)	−9.3 (−15.0 to −3.5)
Relative index of inequality (Q5:Q1)	0.3 (0.1–0.5)	0.3 (0.1–0.5)
Concentration index	−0.2 (−0.3 to −0.1)	−0.2 (−0.3 to −0.1)

*CI* Confidence interval

### Determinants of financial hardship

Table 3 presents the results from the Poisson regression model of risk factors for catastrophic health payment, impoverishment, and distress financing. Household consumption quintile was inversely associated with all three financial hardship indicators. Households in the poorest quintile were more likely to incur catastrophic payment, impoverishment and distress financing than the richest quintile. Utilization of health services was also significantly associated with three financial hardship indicators, with those using inpatient care services having the largest relative risk. The presence of a member with chronic illness in a household increased the risk of impoverishment by a factor of 1.9 (95% CI: 1.1–3.4).

**Table 3:** Multiple Poisson regression model for financial hardship indicators, Bangladesh, 2011

Variable	Relative risk (95% confidence interval)		
	Catastrophic payment	Impoverishment	Borrowing or selling
Average number of children in HH	1.12 (1.02–1.23)	1.04 (0.9–1.19)	1.01 (0.89–1.14)
Average number of adult in HH	1.39 (1.03–1.87)	1.25 (0.92–1.69)	1.38 (1.03–1.85)
Member with chronic disease			
Yes	1.41 (0.87–2.29)	1.90 (1.08–3.36)	1.70 (0.87–3.31)
No	1.00	1.00	1.00
HH member over 65 years			
Yes	1.22 (0.79–1.90)	1.11 (0.53–2.34)	1.50 (0.81–2.79)
No	1.00	1.00	1.00
HH consumption quintile			
Quintile 1 (poorest)	4.26 (1.67–10.88)	17.34 (3.73–80.55)	4.03 (1.72–9.45)
Quintile 2	2.84 (1.15–7.05)	5.19 (1.14–23.71)	2.23 (0.91–5.45)
Quintile 3	2.65 (1.25–5.59)	3.11 (0.92–10.49)	1.57 (0.64–3.83)
Quintile 4	2.28 (1.12–4.65)	2.71 (0.98–7.52)	1.39 (0.62–3.12)
Quintile 5 (richest)	1.00	1.00	1.00
Household size	0.98 (0.88–1.09)	1.03 (0.89–1.19)	1.07 (0.95–1.21)
Care-seeking behavior			
Inpatient	6.67 (4.50–9.90)	7.09 (3.68–13.65)	3.95 (2.52–6.18)
Outpatient public	0.67 (0.40–1.13)	0.99 (0.51–1.9)	0.60 (0.32–1.11)
Outpatient private	1.00	1.00	1.00
Outpatient public and private	1.58 (0.93–2.68)	1.35 (0.63–2.9)	1.59 (0.78–3.23)
Self-medication/traditional healer	0.24 (0.13–0.44)	0.35 (0.17–0.69)	0.23 (0.12–0.44)
Household head education			
No education	2.46 (1.34–4.53)	1.40 (0.57–3.45)	1.60 (0.72–3.53)
Primary	1.71 (0.92–3.17)	1.14 (0.48–2.7)	1.78 (0.83–3.82)
Secondary	1.39 (0.81–2.39)	0.61 (0.25–1.47)	1.29 (0.62–2.66)
Higher	1.00	1.00	1.00

*HH* household

## Discussion

This study found residents in Bangladesh faced serious problems with healthcare financing. This is the first study in Bangladesh to include evidence in health financing research regarding inequalities in UHC financial risk protection indicators. From this study, we found that around one in ten household incurred financial catastrophe, and one in 20 non-poor households became poor due to healthcare costs. Poor households spent less on healthcare, facing disproportionately higher financial burden.

On average, households spent about 11.0% of their total household budget on healthcare, and had high incidence of financial hardship as a result of OOP healthcare payments. The study demonstrated that the overall rate of impoverishment was 5.6%. Similar rates of impoverishment was found in China (5.7%) and Vietnam (7.7%) [31]. Our study also found that around 7% of households faced distress financing (borrowing or selling household assets) to pay for healthcare costs. Consistent with other studies in developing nations [19, 27, 32], the risk of using distress financing was strongly associated with household socio-economic status. Financial hardship including catastrophic payment and distress financing from healthcare were substantially high in the poorest households compared to their richest counterparts, and this is consistent with previous studies from developing countries [31, 33, 34].

Financial hardship is closely linked with the utilization of health services in Bangladesh. In this study, incidence of financial hardship for inpatient care was quite different from those that received care in outpatient facilities. For example, inpatient treatment costs incurred around 69% of financial catastrophe, 41% of impoverishment, and 37% of distress financing, while the proportion was nearly four times lower among public and private outpatient care. These findings were similar to several studies from developing countries [19, 27]. According to Xu and colleagues [25], the availability of health services requiring OOP payments, low ability to pay, and absence of health insurance are the three key preconditions for financial risk including catastrophic payments, impoverishment, or distress financing. We found that all these conditions were present in our study area. Therefore, it is clear that public health services fail to perform their social safety net roles properly.

The higher burden of financial hardship found in this study proved that health financing in Bangladesh relies heavily on OOP payments for both public and private health services. A previous study suggested that although about 70% of households in Bangladesh received inpatient treatment from public facilities, but were more likely to receive outpatient treatment in private health services [35]. Subsidized public health services in Bangladesh may be associated with financial risk because of unofficial charges, tips, lack of monitoring systems in subsidized programs, and dependency on private health markets for essential ancillary services such as medical supplies and drugs [35]. For instance, a study in Bangladesh reported that the average level of per-patient unofficial fees was 12 times the amount that could be expected in official payments – assuming that no respondents were exempted from paying official fees [36].

In Bangladesh, although health has been prioritized since the inception of the First Five Year Plan (1973–1978), for the first four decades after independence, the country lacked a national health financing policy to reduce the burden of financial hardship caused by OOP health payment. Bangladesh’s first 20 years health care financing strategy, developed in 2012, had a vision to halve the share of OOP payment in total health expenditure and to implement a social health protection scheme by 2032 through raising tax revenue and mandatory social health contribution [37]. However, the implementation of social protection scheme remained a challenge since majority of the people are engaged in informal sectors. The government of Bangladesh aims to increase the allocation of budget for health to 15% by 2032 from its current level of about 5%, which might be challenging. The government recently implemented a pilot project of health insurance, called *Shastyo Suroksha Karmasuchi* (SSK), for the population of three sub-districts of Dhaka division living below the poverty line. The benefit package includes one health card for each household and free treatment services for 50 diseases. The per capita health expenditure in Bangladesh has been increasing over the years, from 9.1 US dollars in 2000 to 30.8 US dollars by 2014. The benefit package might be insufficient to protect poor households from the burden of OOP payment considering the ever-increasing health expenditure. The reduction of OOP payment burden will be difficult unless national health insurance scheme is to be implemented to cover all citizens with priority for the poor population. A nationwide implementation of health scheme with better benefit package for total population like Universal Coverage Scheme of Thailand or *Seguro Popular* of Mexico can protect people from this high burden of OOP payment [38, 39]. Through the introduction of risk pooling mechanisms, many other low-, middle- and high-income countries have successfully reduced user fees at the point of care and mitigated the economic risk that OOP payments posed for families [33, 40].

### Strength and limitations

The research protocol and sampling process in this study was designed carefully to avoid any bias in the results. Despite this, the study has some limitations. First, the study was conducted only in urban populations from one metropolitan area of the country; therefore the results cannot necessarily be generalized to the whole country. However, we selected our study subjects through a random selection process to improve the representative nature of the sample which may be applicable to other urban areas in Bangladesh. Second, our study was cross-sectional, leading to its inability to capture seasonal variations in household consumption or illness-related expenditure and coping strategies. Third, a relatively small number of households experienced inpatient hospitalization in the past 30 days recall period so we were unable to provide any result separately for inpatient public versus inpatient private facilities.

## Conclusion

The study clearly revealed that the existing health financing system in Bangladesh fails to protect households from financial risk associated with health service. Therefore, health financing reform is essential to protect people from financial shocks caused by OOP payment. Reforms should include increasing government spending on health through budget reallocation, proper monitoring of subsidized programs, ensuring standard costs for both official and unofficial fees across all public facilities, and committing to health insurance for the whole population.

## Additional file

Additional file 1: Table S1. Basic characteristics of households, Bangladesh, 2011. **Table S2.** Incidence of catastrophic expenditure, impoverishment and distress financing by care-seeking behavior and household characteristics, Bangladesh, 2011. (DOC 62 kb)

## Abbreviations

CI: Confidence interval
GDP: Gross domestic product
NGO: Non-Governmental Organization
OOP: Out-of-pocket
RII: Relative index of inequality
SDGs: Sustainable development goals
SII: Slope index of inequality
SSK: Shastyo Suroksha Karmasuchi
UHC: Universal health coverage
WB: World Bank
WHO: World Health Organization
