# Supplementary material for: Inequalities in financial risk protection in Bangladesh: an assessment of universal health coverage
Source: Int J Equity Health. 2017 Apr 4;16:59. doi: 10.1186/s12939-017-0556-4 (PMC5381038; doi:10.1186/s12939-017-0556-4)
Supplement: Additional file 1: Table S1. — Basic characteristics of households, Bangladesh, 2011. Table S2. Incidence of catastrophic expenditure, impoverishment and distress financing by care-seeking behavior and household characteristics, Bangladesh, 2011. (DOC 62 kb) [file 12939_2017_556_MOESM1_ESM.doc]

**Additional file 1**

**Table S1. Basic characteristics of households**, Bangladesh, 2011

| **Characteristics** | **Mean (95% confidence interval)** |
| --- | --- |
| Average household size | 4.6 (4.5–4.7) |
| Average number of dependent members per household | 2.0 (2.0-2.1) |
| Average number of illnesses per household | 2.8 (2.6-2.9) |

Note: Dependent members include household members aged 15 years or lower, 65 years and older, student or disabled person.

**Table S2. Incidence of catastrophic expenditure, impoverishment and distress financing by care-seeking behavior** and household characteristics, Bangladesh, 2011

| Characteristics | Frequency (n = 1593) | Catastrophic payments | Impoverishment  (95% CI) | Distress financing |
| --- | --- | --- | --- | --- |
| **Care-seeking behavior** |  |  |  |  |
| Inpatient | 65 | 68.5 (56.6-78.4) | 40.9 (30.1–52.7) | 37.0 (25.1-50.8) |
| Outpatient public | 253 | 9.0 (5.6-14.2) | 7.4 (4.9–10.9) | 6.8 (4.0-11.1) |
| Outpatient private | 385 | 9.3 (6.5-13.1) | 4.7 (2.7–8.1) | 8.3 (5.5-12.3) |
| Outpatient public and private | 105 | 16.9 (10.2-26.8) | 6.1 (2.9–12.6) | 8.3 (5.5-12.3) |
| Self-medication/traditional healer | 785 | 2.8 (1.6-4.8) | 2.5 (1.5–4.1) | 14.1 (7.7-24.4) |
| **Educational status of household head** |  |  |  |  |
| No education | 258 | 15.2 (11.1-20.5) | 11.6 (8.2–16.3) | 9.4 (5.6-15.5) |
| Primary | 310 | 11.4 (7.8-16.6) | 8.7 (6.2–2.1) | 10.5 (7.1-15.2) |
| Secondary | 420 | 7.2 (5.0-10.2) | 3.0 (1.7–5.0) | 5.8 (3.6-9.2) |
| Higher | 605 | 5.9 (4.1-8.4) | 3.0 (1.8–4.7) | 4.6 (2.8-7.3) |
| Household member over 65 years |  |  |  |  |
| Yes | 136 | 11.0 (6.4-18.3) | 6.3 (3.2–12.1) | 10.5 (5.7-18.8) |
| No | 1457 | 8.8 (7.0-11.0) | 5.6 (4.4–7.0) | 6.7 (5.0-8.9) |
| Member with chronic disease |  |  |  |  |
| Yes | 1148 | 10.5 (8.3-13.3) | 6.6 (5.3–8.2) | 8.0 (5.9-10.8) |
| No | 445 | 5.2 (3.1-8.5) | 3.2 (2.0–5.3) | 3.8 (2.0-7.0) |
| Household consumption quintile |  |  |  |  |
| Quintile 1(poorest) | 319 | 14.3 (10.3-19.6) | 15.1 (11.3–19.8) | 11.7 (8.3-16.4) |
| Quintile 2 | 319 | 9.7 (6.2-15.0) | 4.6 (2.6–7.9) | 7.6 (4.9-11.7) |
| Quintile 3 | 318 | 9.2 (5.7-14.5) | 3.2 (1.7–6.0) | 6.1 (3.1-11.8) |
| Quintile 4 | 319 | 7.1 (4.3-11.4) | 2.9 (1.2–6.8) | 4.8 (2.6-8.6) |
| Quintile 5 (richest) | 318 | 3.4 (1.7-6.4) | 1.2 (0.5–3.1) | 3.9 (2.0-7.7) |

CI: Confidence interval
